# Long-Term Surveillance of Antibiotic Prescriptions and the Prevalence of Antimicrobial Resistance in Non-Fermenting Gram-Negative Bacilli

**DOI:** 10.3390/microorganisms8030397

**Published:** 2020-03-12

**Authors:** Chia-Huei Chou, Yi-Ru Lai, Chih-Yu Chi, Mao-Wang Ho, Chao-Ling Chen, Wei-Chih Liao, Cheng-Mao Ho, Yu-An Chen, Chih-Yu Chen, Yu-Tzu Lin, Chia-Der Lin, Chih-Ho Lai

**Affiliations:** 1 Departments of Infectious Disease, China Medical University Hospital, Taichung 40447, Taiwan; 2 School of Medicine, Department of Medical Laboratory Science and Biotechnology, Graduate Institute of Biomedical Sciences, China Medical University, Taichung 40402, Taiwanyuzi7676@hotmail.com (Y.-T.L.); 3 Graduate Institute of Biomedical Sciences, Department of Microbiology and Immunology, Chang Gung University, Taoyuan 33302, Taiwan; 4 Department of Pharmacology and Toxicology, Virginia Commonwealth University, Richmond, VA 23284, USA; 5 Department of Pulmonary and Critical Care Medicine, China Medical University Hospital, Taichung 40447, Taiwan; cychen0808@gmail.com; 6 Department of Laboratory Medicine and Clinical Pathology, Taichung Tzu Chi Hospital, Buddhist Tzu Chi Medical Foundation, Taichung 42743, Taiwan; shihkuo.ho@msa.hinet.net; 7 Department of Nursing, Hungkuang University, Taichung 43302, Taiwan; 8 Department of Life Sciences, National Chung Hsing University, Taichung 40227, Taiwan; yachen.cmu@gmail.com; 9 Department of Urology, University of Texas Southwestern Medical Center, Dallas, TX 75390, USA; 10 Department of Otolaryngology-Head and Neck Surgery, China Medical University and Hospital, Taichung 40447, Taiwan; 11 Department of Nursing, Asia University, Taichung 41354, Taiwan; 12 Molecular Infectious Disease Research Center, Department of Pediatrics, Chang Gung Memorial Hospital, Linkou 33305, Taiwan

**Keywords:** antimicrobial prescription, antimicrobial resistance, *Acinetobacter calcoaceticus-Acinetobacter baumannii* complex, *Pseudomonas aeruginosa*

## Abstract

The increasing emergence of multidrug-resistant (MDR) bacteria has been recognized as a public health threat worldwide. Hospitalized patients and outpatients are commonly infected by non-fermenting Gram-negative bacilli (NFGNB), particularly the *Acinetobacter calcoaceticus*-*Acinetobacter baumannii* complex (ACB) and *Pseudomonas aeruginosa*. Antimicrobial agents are critical for treating the nosocomial infections caused by NFGNB. The aim of this study was to assess antimicrobial resistance and the use of antimicrobial agents. The bacterial isolates of 638,152 specimens from both inpatients and outpatients, retrieved from 2001 to 2008 at a medical center in central Taiwan, were examined for their susceptibility to various antimicrobial agents, including cefepime, imipenem, ciprofloxacin, gentamicin, amikacin, meropenem, and levofloxacin. Administrated prescriptions of the monitored antibiotics were analyzed using the Taiwan National Health Insurance Research Database (NHIRD). Our results show that the defined daily doses (DDDs) for cefepime, imipenem, and ciprofloxacin increased with time, and a trend toward reduced antimicrobial sensitivities of both ACB and *P. aeruginosa* was noticeable. In conclusion, the antimicrobial sensitivities of ACB and *P. aeruginosa* were reduced with the increased use of antibiotics. Continuous surveillance of antibiotic prescriptions and the prevalence of emerging resistance in nosocomial infections is warranted.

## 1. Introduction

*Acinetobacter calcoaceticus-Acinetobacter baumannii* complex (ACB) and *Pseudomonas aeruginosa* belong to the non-fermenting Gram-negative bacilli (NFGNB) group of bacilli, which cause various nosocomial infections, including pneumonia, septicemia, wound infections, meningitis, and urinary tract infections [1,2]. Antibiotic therapy is the most common and effective way for combating these two NFGNBs; however, their extensive use can lead to increased numbers of antimicrobial resistant bacteria [3,4,5]. This development makes the treatment of patients infected with these pathogens difficult, and consequently increases morbidity and mortality [1,6]. Notably, although carbapenems have been used as the main antimicrobial therapy for multidrug-resistant (MDR)-ACB and MDR-*P. aeruginosa* [7,8], carbapenem resistance has also emerged in these two bacterial species and caused severe nosocomial outbreaks [9,10,11]. These lines of evidence indicate that an increase in antibiotic prescription is closely related to elevated antimicrobial resistance rates.

Correlations between antibiotic use and resistance have been investigated, and the use of certain antibiotics have been found to have stronger positive correlations with the development of resistance than others [12]. Interestingly, extensive use of antibiotics, as opposed to intensive use (for patients with critical need), is more closely related to population-wide antimicrobial resistance [13]. In addition, the rapid spread of resistance mechanisms, and limited treatment options available for resistant pathogens, are becoming fatal factors for infected patients [14]. Accordingly, antibiotic resistance in recent decades has become a worldwide public health challenge, and this is a major consideration in threatening human health globally.

Proper selection and use of antimicrobial agents remain a critical issue in such cases, since antimicrobial resistance has not only become a substantial threat to patients, but also presents a therapeutic challenge, leaving clinicians with barely any options to apply when faced with such a condition [15,16,17]. Hence, there is an urgent need to acquire long-term surveillance data presenting the connection between antibiotic consumption and bacterial resistance. 

In 1995, the Taiwan government established a national health insurance system which is administrated by the Ministry of Health and Welfare [18]. To restrict the use of antimicrobial agents in patients with acute upper respiratory infections, the Taiwan National Health Insurance (BNHI) established a new policy, which dictates that without evidence of bacterial infection, the insurance costs of antibiotics are not reimbursed [19]. Noticeably, this particular act effectively reduced the unnecessary use of antibiotics in patients with acute upper respiratory infections, and decreased the antimicrobial resistance of certain bacterial species [20,21,22,23]. However, the relationship between the use of antimicrobial agents and antimicrobial resistance in nosocomial infections caused by NFGNB remains to be investigated.

The aim of this study was to analyze the antimicrobial resistance of NFGNB, including ACB and *P. aeruginosa* isolated from both hospitalized patients and outpatients, and to examine the correlation with antibiotic use over the period from 2001 to 2008. We further evaluated the impact of antimicrobial prescriptions on the emergence of antimicrobial resistance in ACB and *P. aeruginosa*. The results from this study provide valuable information for physicians to determine appropriate intervention measures.

## 2. Materials and Methods

### 2.1. Study Design and Patient Selection

**Figure 1:**
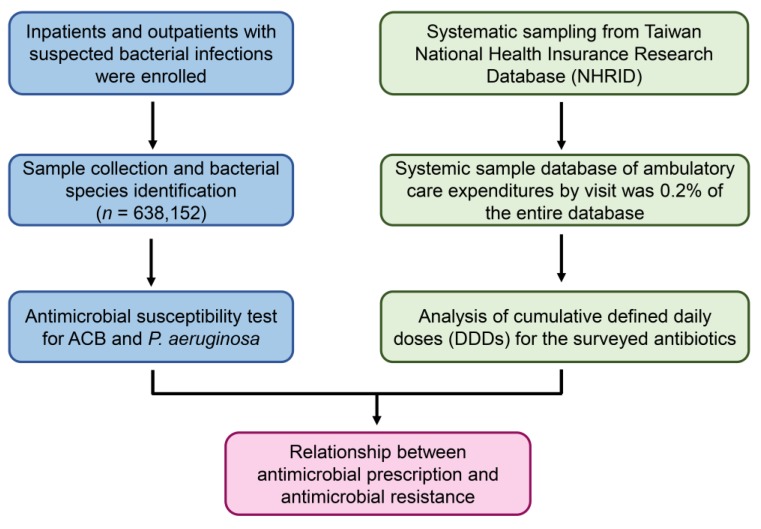
Flowchart of sample collection, antimicrobial determination, and defined daily doses (DDD) analysis. *Acinetobacter calcoaceticus-Acinetobacter baumannii* complex (ACB) and *P. aeruginosa* were isolated from the hospitalized patients and outpatients, and the antimicrobial susceptibility was identified. Cumulative DDDs were analyzed by using National Health Insurance Research Database (NHIRD).

The bacterial isolates of 638,152 specimens were collected from inpatients and outpatients between 1 January 2001 and 31 December 2008 at China Medical University Hospital, Taichung, Taiwan (a 2500-bed tertiary teaching hospital in central Taiwan). The clinical data, which included sex, age, diagnosis, medication, hospital ward, and medical procedures, were recorded for each patient. Bacterial cultures were established from various specimens, including blood, sputum, pus, urine, cerebrospinal fluid (CSF), pleural, ascites, bile, body fluid, and central catheter tips. Excluded were specimens such as stools, throat swabs, and catheter tips that did not meet the criteria of central catheter infection. In addition, some special pathogens were excluded, i.e., *Nocardia* species, *Actinomyces* species, and all fungi. Specimens from patients who did not provide consent, or who were referred to other hospitals, were also excluded (Figure 1). All enrolled patients provided informed consent before beginning the experimental protocol. Clinical and demographic data were collected by medical chart review for the patients with positive culture of ACB or *P. aeruginosa*.

### 2.2. Bacterial Cultures and Identifications

Sample preparation followed the protocol described previously [11,24]. Each isolated sample was collected into a sterile tube or container (except blood culture samples, which were inoculated immediately in blood culture-specific bottles (BD BACTEC^TM^; Becton Dickinson, Franklin Lakes, NJ, USA), sent to the laboratory within two to three hours of collection, and maintained at room temperature. Samples that were not inoculated immediately during a non-working period were stored at room temperature, except urine and CSF, which were maintained at 4 °C and 35 °C, respectively. All bacterial culture samples were inoculated within 24 h of isolation. For sputum cultures, initial Gram stains for quality scores (<10 squamous epithelial cells per low power field) were checked before reporting final results. For urine samples, quantitative cultures using sterile loops were established, and the reporting criteria were >10^5^ and >10^3^ CFU for midstream and indwelling catheters, respectively. All specimens retrieved from patients were streaked across Trypticase soy agar with 5% sheep blood (TSA II)/Levine eosin methylene blue EMB agar (Becton Dickinson) and incubated at 37 °C. Bacterial isolates were identified as ACB and *P. aeruginosa* by use of an automated microbiologic analysis system (BD Phoenix; Becton Dickinson) [10].

### 2.3. Antimicrobial Susceptibility Test

The antimicrobial susceptibility of the bacterial isolates to various antimicrobial agents was determined using a BD Phoenix™ Automated Microbiology System (Becton Dickinson), as described previously [10]. Antimicrobial susceptibility of all the isolates (to cefepime, imipenem, ciprofloxacin, gentamicin, amikacin, meropenem, and levofloxacin) was confirmed by a disk diffusion method, following the guidelines and criteria of the Clinical Laboratory Standards Institute [25]. The definition of MDR was isolates with resistance ≥3 classes of the following antimicrobial agents: antipseudomonal cephalosporins, antipseudomonal carbapenems, β-lactam–β-lactamase inhibitor combinations, antipseudomonal fluoroquinolones, and aminoglycosides [26]. The susceptibility of the ACB and *P. aeruginosa* isolates to various antimicrobial agents was determined by use of the automated system, and these isolates were confirmed to be MDR strains. All MDR strains were stored at −80 °C in bacterial culture broth (Becton Dickinson) containing 20% glycerol, until experimental procedures were done.

### 2.4. Data Source

To examine whether antibiotic use is associated with antimicrobial resistance, a nationwide cohort study was conducted. Data were obtained from the Taiwan National Health Insurance Research Database (NHIRD). The NHIRD was made available for the purpose of research by National Health Research Institutes (NHRI), which managed and analyzed the insurance claims data reported to the Bureau of Health Insurance (BHI) (http://www.nhri.org.tw/nhird/index.htm). For research purposes, the NHRI compiles all medical claims in the National Health Insurance (NHI) program and releases the database annually to the public. Patient consent is not required to access the NHIRD.

### 2.5. Analysis of Antibiotic Prescription from the National Health Insurance Database (NHIRD)

NHIRD-contained medical information, including data on inpatient and outpatient care facilities, drug prescriptions, insurant sex, and date of birth, date of visit, or hospitalization, and diagnoses, is coded in the format of the International Classification of Disease, 9th Revision, Clinical Modification (ICD-9-CM) [27]. A systematic sampling method was used to randomly collect a representative data set from the entire database. The size of the subset from each month was determined by the ratio of the amount of data in each month to that of the entire year. The systematic sampling was then performed for each month to randomly choose a representative subset. The sample database was obtained by combining the subsets from 12 months in each year. The sample database of CD (ambulatory care expenditures by visit) was constructed first, and the relevant observations in OO (details of ambulatory care order) were drawn out as necessary. The systemic sample database of CD was 0.2% of the entire database. All data on the consumption of various antibiotics were obtained from NHRI. Drug code for each analyzed antimicrobial agent was obtained from the Bureau of National Health Insurance. The defined daily doses (DDDs) were recommended by the World Health Organization to assume the average maintenance dose per day of a drug, and analyzed as described previously [28]. The cumulative DDD was calculated by deriving the total prescribed DDD of each antimicrobial agent, namely cefepime (ATC J01DE01), imipenem (ATC J01DH51), ciprofloxacin (ATC J01MA02), gentamicin (ATC J01GB03), amikacin (ATC J01GB06), meropenem (ATC J01DH02), and levofloxacin (ATC J01MA02), for the antibiotic users. This study was approved by the Institutional Review Board of the China Medical University Hospital, Taichung, Taiwan (approval number: CMUH104-REC2-115-CR2; date: 3 July 2017). All the analyzed methods were in accordance with the guidelines approved by the institution.

### 2.6. Statistical Analysis

The antibiotic consumption was analyzed by the cumulative DDDs. Spearman’s rank correlation coefficients were conducted to analyze the association between antibiotic sensitivity (%), years, and DDDs. The results of Spearman’s coefficients (*r*_s_) and *p*-values for the examined antibiotics were reported. A *p*-value of < 0.05 indicated statistical significance. All analyses were conducted using SAS statistical software (Version 9.3 for Windows; SAS Institute, Inc., Cary, NC, USA).

## 3. Results

### 3.1. Antimicrobial Susceptibility of ACB and P. aeruginosa

**Figure 2:**
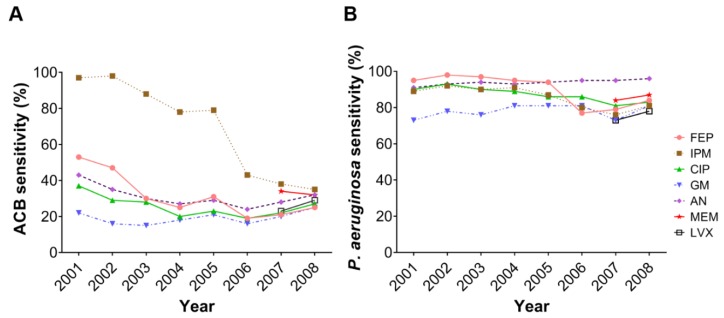
The prevalence of antimicrobial sensitivity for ACB and *P. aeruginosa*. (**A**) ACB and (**B**) *P. aeruginosa* were isolated from inpatients and outpatients in China Medical University Hospital (CMUH) from 2001 to 2008. The prevalence of sensitivity to each antibiotic was analyzed: AN, amikacin; CIP, ciprofloxacin; FEP, cefepime; GM, gentamicin; IPM, imipenem; LVX, levofloxacin; MEM, meropenem.

Initially, the antimicrobial susceptibility of all bacterial isolates was determined by disk diffusion method. As shown in Figure 2A, the sensitivity of ACB to all the antimicrobial agents exhibited declining trends from 2001 to 2008. ACB sensitivity to cefepime (*p* = 0.0248) and imipenem (*p* = 0.0011) was significantly reduced during the study period (Figure S1). The sensitivity of *P. aeruginosa* to antimicrobial agents decreased between 2001 and 2008 (Figure 2B). Notably, a dramatic reduction in antimicrobial sensitivity was retained in *P. aeruginosa* to cefepime (*p* = 0.0211), imipenem (*p* = 0.0368), and ciprofloxacin (*p* = 0.0018).

### 3.2. Relationship between Antimicrobial Prescription and Antimicrobial Resistance

**Figure 3:**
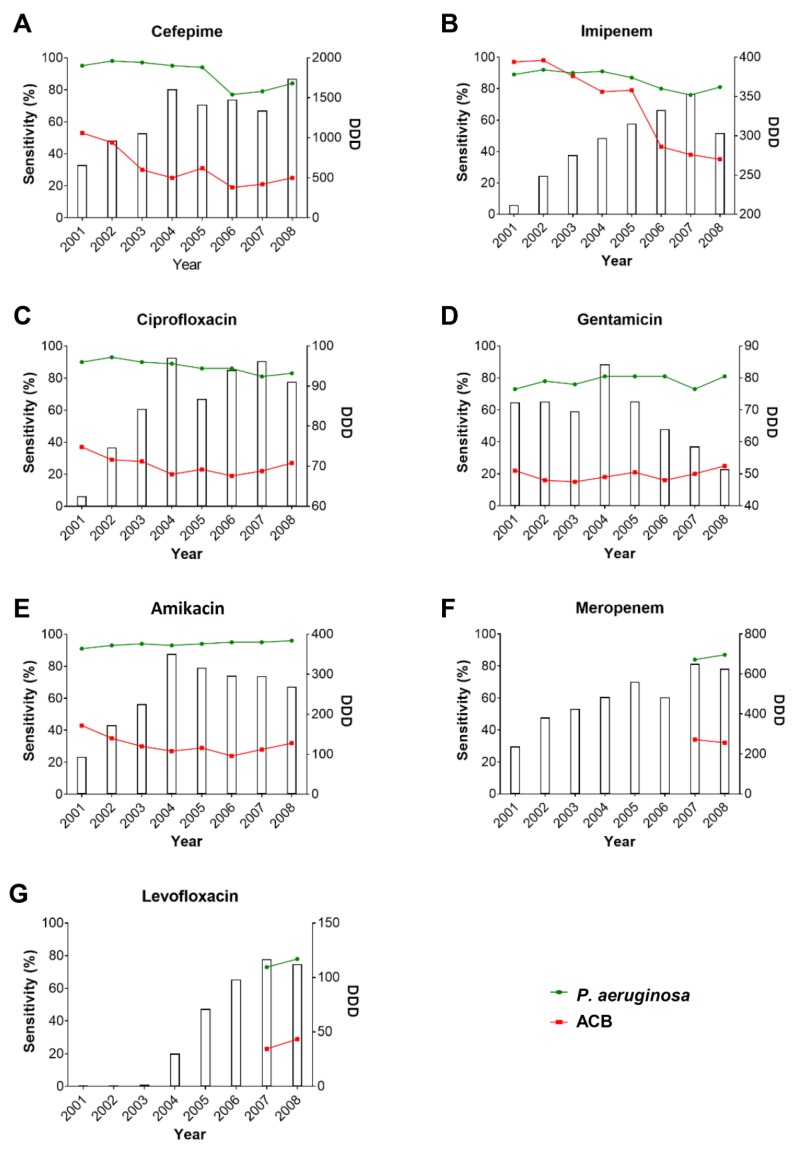
The annual prescriptions of antibiotics and antibiotic susceptibility of ACB and *P. aeruginosa*. Total prescribed DDDs of (**A**) cefepime, (**B**) imipenem, (**C**) ciprofloxacin, (**D**) gentamicin, (**E**) amikacin, (**F**) meropenem, and (**G**) levofloxacin were analyzed from the National Health Insurance Research Database (NHIRD). Bars indicate the annual consumption of antimicrobials (DDDs). Green lines and red lines represent the sensitivity of *P. aeruginosa* and ACB to antimicrobial agents, respectively.

To analyze the consumption of antimicrobial agents for the total population in Taiwan, a representative data set from the NHIRD was conducted and assessed through the systematic sampling approach, as described in the methods section. Figure 3 shows the annual consumption of the surveyed antimicrobial agents in Taiwan from 2001 to 2008. The results reveal that the DDDs for cefepime, imipenem, and ciprofloxacin increased over time; however, the antimicrobial susceptibilities of both ACB and *P. aeruginosa* were reduced. With the DDD increase, the sensitivity of ACB to imipenem (*p* = 0.0279), ciprofloxacin (*p* = 0.0046), and amikacin (*p* = 0.0107), and sensitivity of *P. aeruginosa* to imipenem (*p* = 0.0218) significantly decreased (Figure S2). In parallel, the DDDs for meropenem and levofloxacin increased annually. The consumption of gentamicin and amikacin increased from 2001 to 2004, and then gradually decreased from 2005 to 2008, but the sensitivity of *P. aeruginosa* did not change accordingly.

## 4. Discussion

NFGNB infections are associated with many severe diseases, including septicemia, pneumonia, surgical site infections, and ventilator-associated pneumonia [29]. NFGNB have wide ranging isolation rates (from 2.18% to 45.9%), which have been determined from different clinical samples [29,30,31]. A previous study in India found a significantly high prevalence of NFGNB (11.6%) in clinical samples, the most common of which were *P. aeruginosa* and *A. baumannii* [32]. Notably, a 10-year epidemiological study in Hungary recently reported that a high proportion of NFGNB were MDR strains: 9.66% were *Acinetobacter* spp. and 8.54% were *Pseudomonas* spp. [33]. The high intrinsic resistance of NFGNB to antimicrobials makes the treatment of infections caused by them difficult and expensive [31]. As *P. aeruginosa* and ACB are the most predominantly isolated NFGNB, and MDR among them is a major concern, it is crucial to build a well-functioning system to efficiently cope with any upcoming antimicrobial resistance crisis.

The results of the current study show that DDDs for cefepime, imipenem, and ciprofloxacin increased over time. Conversely, the trend of antimicrobial sensitivity to both ACB and *P. aeruginosa* decreased over time. Our results demonstrate that antimicrobial sensitivity to ACB and *P. aeruginosa* isolated from patients is reduced with an increase in antibiotic consumption. Moreover, these findings are consistent with those of previous studies conducted in other countries [34,35,36].

Taiwan has established a national health insurance system that has been administered by the Ministry of Health and Welfare since 1995 [18]. Consequently, many first-line antibiotics have become considerably more affordable; however, it has also led to unnecessary and excessive use of antibiotics. Starting from February 2001, due to growing concerns regarding antibiotic abuse, the Bureau of National Health Insurance has halted the reimbursement for antimicrobial-based treatment of acute upper respiratory infections, if no evidence of bacterial involvement is available [19]. This specific action has successfully reduced antibiotic consumption and attenuated the rapid appearance of antimicrobial resistance [20,21,22,23]. Therefore, appropriate antibiotic stewardship strategies are of considerable importance in controlling the development of antibacterial resistance, as has been demonstrated in multiple countries, including Taiwan [34,35,36,37,38,39,40,41].

In the current study, we surveyed the prescriptions of seven antimicrobial agents over a period of eight years, and found, in general, an increased usage of all monitored antibiotics, except two aminoglycoside drugs, gentamicin and amikacin, which showed a decreasing tendency in consumption from 2005 to 2008. Consistently, reduced susceptibility of ACB to all antibiotics was observed, with the most considerable decline involving cefepime and imipenem. Nevertheless, a relatively slight change in antimicrobial susceptibility was seen in *P. aeruginosa*. Coincidentally, resistance toward cefepime and imipenem in *P. aeruginosa* also presented the most noticeable growth among the other antibiotics.

Although there were certain pre-existing limitations in accessing the complete data on meropenem resistance, we still observed a positive association between the use of carbapenems (including cefepime, imipenem, and meropenem) and carbapenem resistance in ACB and *P. aeruginosa*. It has been reported that treatment with carbapenems is often an effective strategy against ACB and *P. aeruginosa* infections; consequently, the clinical use of carbapenems, particularly in ICUs, is worryingly high [42,43]. This might explain the wild emergence rate of carbapenem resistance. Although the use of amikacin increased markedly between 2001 and 2004, the sensitivity trends of ACB and *P. aeruginosa* were comparatively more stable than those of the other monitored antibiotics.

The other tracked aminoglycoside, gentamicin, was not prescribed as often as amikacin, potentially because it exhibits comparatively higher nephrotoxicity and lower effects on bacteria [35]. Nonetheless, the sensitivity trends of both the bacterial species remained considerably steady. Moreover, we found that both the bacteria can resuscitate the antimicrobial activity of the two aminoglycoside antibiotics (amikacin and gentamicin) that we have monitored over three years (2006–2008). It is rational to assume that the increase in the usage of an antibiotic would lead to the prevalence of its resistance, and vice versa. However, the inverse situation is infrequently described, which might be attributed to the existence of other interactive factors that have not been considered.

The Taiwan government has launched an NHI program that has provided comprehensive coverage for all residents in the country for decades [18]. All insurance claims are reviewed by medical reimbursement experts and recorded in the NHI database, which enabled the appropriate selection of patients to represent the underlying population. Accordingly, by analyzing the data from the NHIRD, we have previously performed some studies and investigated the association of several drugs with numerous diseases [21,28,44,45]. Therefore, the utilization of nationwide data to analyze the consumption of various antibiotics in this study is reliable.

The antimicrobial stewardship program has changed since 2009 in our hospital. Before 2009, an infectious disease specialist and physician would agree to the use of antibiotics. In 2009, the hospital developed a computer program that includes more detailed clinical data, including symptoms, signs, and laboratory diagnoses of patients via an antibiotic agreement system. In this new policy, the infectious disease specialists agree to antibiotic use after thoroughly reviewing the patient’s information. Currently, the association between antibiotic prescriptions and the prevalence of antimicrobial resistance from 2009 to 2019 is under investigation. The results of the comparison of antimicrobial resistance in NFGNB before and after the changes to the antimicrobial stewardship program in the hospital will be reported in the near future.

## 5. Conclusions

This study showed that DDDs for cefepime, imipenem, and ciprofloxacin increased over time, while the trend of antimicrobial sensitivity to both ACB and *P. aeruginosa* decreased. The overuse of antimicrobial agents has led to a rather high therapy failure rate, for which the increased antimicrobial resistance is to be blamed. Further efforts are required for the surveillance of various treatment options, since the analytical reports consisting of the retrospective research have shown significance in assessing the efficacy of different interventions. Moreover, an effective antibiotic stewardship strategy would be beneficial for controlling antimicrobial resistance of ACB and *P. aeruginosa* infections in patients. The information provided in this study may aid in the selection of an appropriate empirical regimen for antibiotic treatments.

## Supplementary Materials

The following are available online at https://www.mdpi.com/2076-2607/8/3/397/s1.
