# Supplementary material for: Long-Term Surveillance of Antibiotic Prescriptions and the Prevalence of Antimicrobial Resistance in Non-Fermenting Gram-Negative Bacilli
Source: Microorganisms. 2020 Mar 12;8(3):397. doi: 10.3390/microorganisms8030397 (PMC7142802; doi:10.3390/microorganisms8030397)
Supplement: Supplementary file 1 [file microorganisms-08-00397-s001.pdf]

Supplementary Materials

Long-Term Surveillance of Antibiotic Prescriptions and the Prevalence of Antimicrobial Resistance in Non-Fermenting Gram-Negative Bacilli

Chia-Huei Chou [1,2,†], Yi-Ru Lai [3,†], Chih-Yu Chi [1,2], Mao-Wang Ho [1,2], Chao-Ling Chen [4], Wei-Chih Liao [2,5], Cheng-Mao Ho [6,7], Yu-An Chen [8,9], Chih-Yu Chen [5], Yu-Tzu Lin [2], Chia-Der Lin [2,10,*] and Chih-Ho Lai [2,3,9,11,12,*]

[1] Departments of Infectious Disease, China Medical University Hospital, Taichung 40447, Taiwan; c3716@ms32.hinet.net (C.-H.C.); cychyi@gmail.com (C.-Y.C.); D7905@mail.cmuh.org.tw (M.-W.H.)
[2] School of Medicine, Department of Medical Laboratory Science and Biotechnology, Graduate Institute of Biomedical Sciences, China Medical University, Taichung 40402, Taiwan; weichih.liao@mail.cmu.edu.tw (W.-C.L.); yuzi7676@hotmail.com (Y.-T.L.)
[3] Graduate Institute of Biomedical Sciences, Department of Microbiology and Immunology, Chang Gung University, Taoyuan 33302, Taiwan; d0801202@cgu.edu.tw
[4] Department of Pharmacology and Toxicology, Virginia Commonwealth University, Richmond, VA 23284, USA; lindaswedo@gmail.com
[5] Department of Pulmonary and Critical Care Medicine, China Medical University Hospital, Taichung 40447, Taiwan; cychen0808@gmail.com (C.-Y.C.)
[6] Department of Laboratory Medicine and Clinical Pathology, Taichung Tzu Chi Hospital, Buddhist Tzu Chi Medical Foundation, Taichung 42743, Taiwan; shihkuo.ho@msa.hinet.net (C.-M.H.)
[7] Department of Nursing, Hungkuang University, Taichung 43302, Taiwan
[8] Department of Life Sciences, National Chung Hsing University, Taichung 40227, Taiwan; yachen.cmu@gmail.com (Y.-A.C.)
[9] Department of Urology, University of Texas Southwestern Medical Center, Dallas, TX 75390, USA
[10] Department of Otolaryngology-Head and Neck Surgery, China Medical University and Hospital, Taichung 40447, Taiwan
[11] Department of Nursing, Asia University, Taichung 41354, Taiwan
[12] Molecular Infectious Disease Research Center, Department of Pediatrics, Chang Gung Memorial Hospital, Linkou 33305, Taiwan
[*] Correspondence: chlai@mail.cgu.edu.tw (C.-H.L.); d6355@mail.cmuh.org.tw (C.-D.L); Tel.: +886-3-2118800 ext. 5116 (C.-H.L.); +886-4-22053366 ext. 2101 (C.-D.L);
[†] These authors contributed equally to this work.

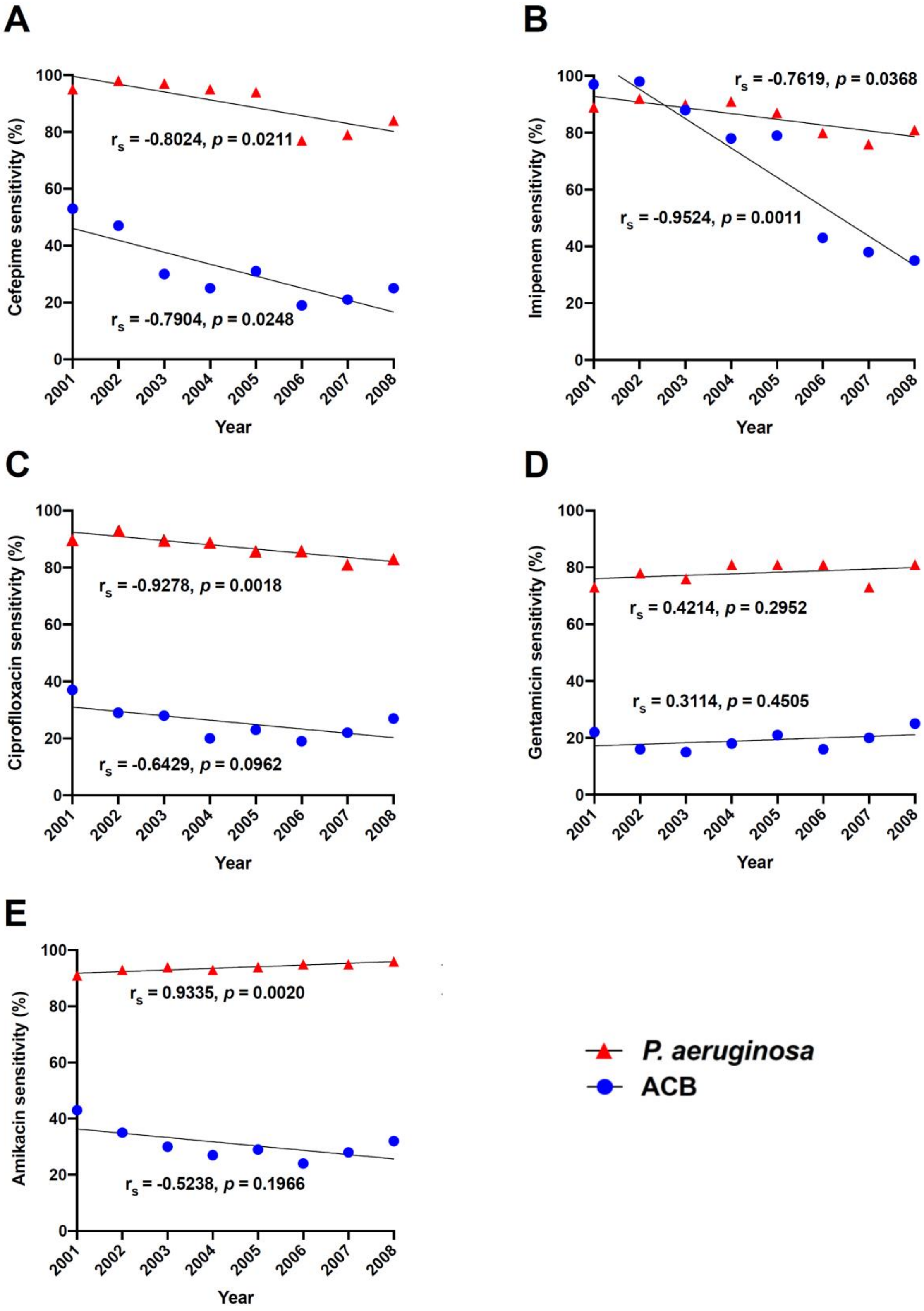

Figure S1. The correlation between antimicrobial sensitivity (%) and years. Antimicrobial sensitivities (%) of *P. aeruginosa* and ACB to (A) cefepime, (B) imipenem, (C) ciprofloxacin, (D) gentamicin, and (E) amikacin were analyzed. *p*-value of Spearman rank correlation test, and r_s = Spearman correlation coefficient. $p < 0.05$ was considered statistically significant. *P. aeruginosa*, *Pseudomonas aeruginosa;* ACB, *Acinetobacter calcoaceticus-Acinetobacter baumannii* complex.

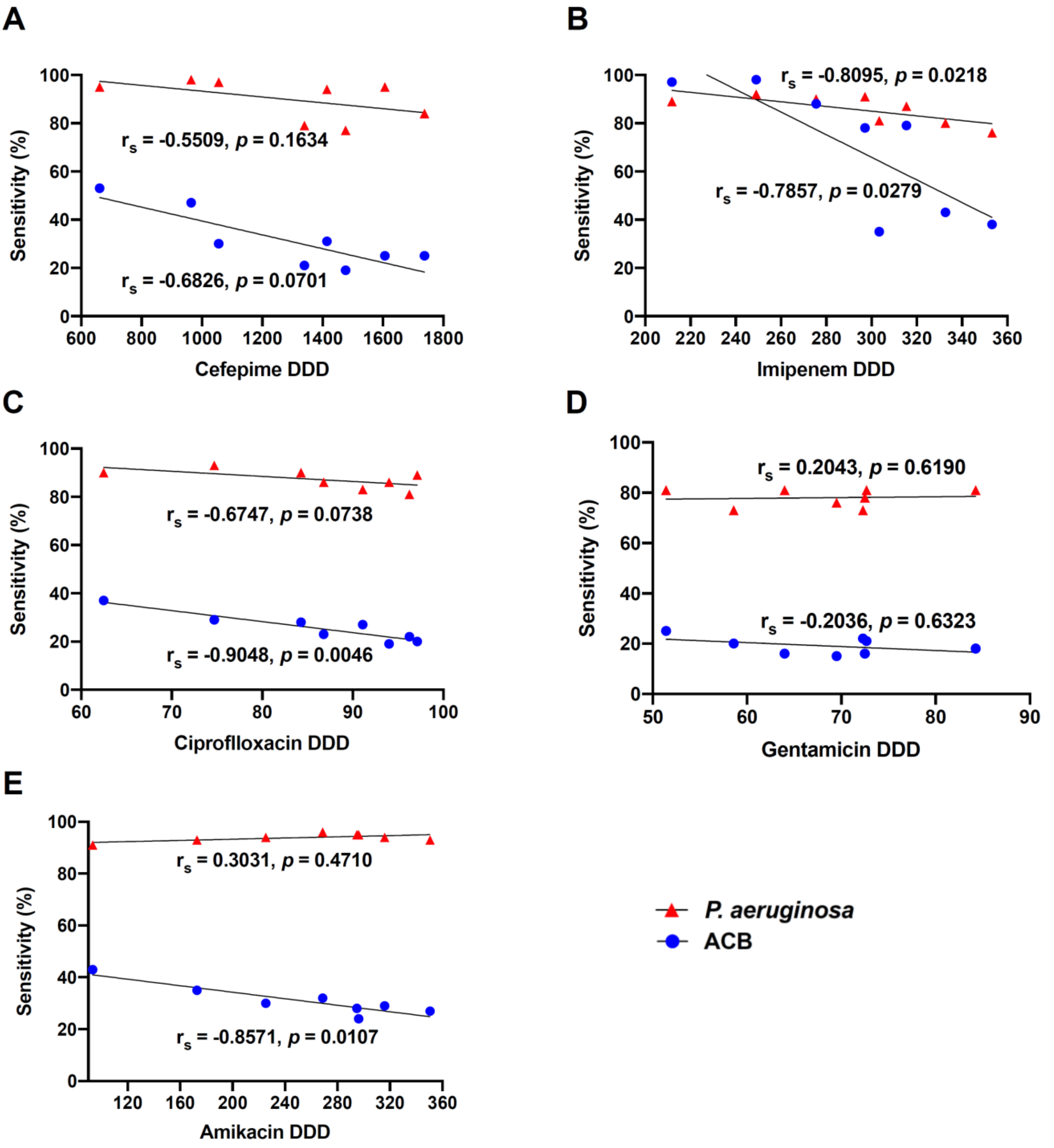

Figure S2. The correlation between antimicrobial sensitivity (%) and DDDs. Antimicrobial sensitivities (%) of *P. aeruginosa* and ACB to (A) cefepime, (B) imipenem, (C) ciprofloxacin, (D) gentamicin, and (E) amikacin were analyzed. Total prescribed DDDs of the antibiotics were analyzed from NHIRD. *p*-value of Spearman rank correlation test, and r_s = Spearman correlation coefficient. $p < 0.05$ was considered statistically significant. DDDs: defined daily doses; *P. aeruginosa*, *Pseudomonas aeruginosa*; ACB, *Acinetobacter calcoaceticus-Acinetobacter baumannii* complex.
